# Doxycycline PEP can induce doxycycline resistance in *Klebsiella pneumoniae* in a *Galleria mellonella* model of PEP

**DOI:** 10.3389/fmicb.2023.1208014

**Published:** 2023-08-30

**Authors:** Chris Kenyon, Zina Gestels, Thibaut Vanbaelen, Said Abdellati, Dorien Van Den Bossche, Irith De Baetselier, Basil Britto Xavier, Sheeba Santhini Manoharan-Basil

**Affiliations:** ^1^STI Unit, Department of Clinical Sciences, Institute of Tropical Medicine, Antwerp, Belgium; ^2^Division of Infectious Diseases and HIV Medicine, University of Cape Town, Cape Town, South Africa; ^3^Clinical Reference Laboratory, Department of Clinical Sciences, Institute of Tropical Medicine, Antwerp, Belgium; ^4^Hospital Outbreak Support Team-HOST, Ziekenhuis Netwerk Antwerpen Middelheim, Antwerp, Belgium

**Keywords:** *Neisseria gonorrhoeae*, *Klebsiella pneumoniae*, doxycycline PEP, WGS, *in-vivo* emergence, DoxyPEP, ST220 *Klebsiella pneumoniae*

## Abstract

**Background:**

Four randomized controlled trials have now established that doxycycline post exposure (sex) prophylaxis (PEP) can reduce the incidence of chlamydia and syphilis in men who have sex with men. These studies have concluded that the risk of selecting for antimicrobial resistance is low. We evaluated this risk *in vitro* and *in vivo* using a *Galleria mellonella* infection model.

**Methods:**

We evaluated how long it took for doxycycline resistance to emerge during passage on doxycycline containing agar plates in 4 species – *Escherichia coli*, *Klebsiella pneumoniae*, *Neisseria gonorrhoeae* and *Neisseria subflava*. We then assessed if *K. pneumoniae* could acquire resistance to doxycycline (and cross resistance to other antimicrobials) during intermittent exposure to doxycycline in a *Galleria mellonella* model of doxycycline PEP.

**Results:**

In our passage experiments, we found that resistance first emerged in *K. pneumoniae*. By day 7 the *K. pneumoniae* MIC had increased from 2 mg/L to a median of 96 mg/L (IQR 64–96). Under various simulations of doxycycline PEP in the G. mellonella model, the doxycycline MIC of *K. pneumoniae* increased from 2 mg/L to 48 mg/L (IQR 48–84). Ceftriaxone and ciprofloxacin MICs increased over ten-fold. Whole genome sequencing revealed acquired mutations in ramR which regulates the expression of the AcrAB-TolC efflux pump.

**Conclusion:**

Doxycycline PEP can select for doxycycline, ceftriaxone and ciprofloxacin resistance in *K. pneumoniae* in a G. mellonella model. The emergent ramR mutations were similar to those seen in circulating strains of *K. pneumoniae*. These findings suggest that we need to assess the effect of doxycycline PEP on resistance induction on a broader range of bacterial species than has hitherto been the case.

## Introduction

Four randomized controlled trials have now established that doxycycline can reduce the incidence of chlamydia and syphilis in men who have sex with men (MSM) (Bolan et al., 2015; Molina et al., 2018; Luetkemeyer et al., 2022; Molina et al., 2023). The Doxycycline Post Exposure Prophylaxis (DoxyPEP) study, for example, found that men randomized to consumption of 200 mg of doxycycline within 24 h of every condomless sex act had an approximately 60% lower incidence of chlamydia, syphilis and gonorrhea (Luetkemeyer et al., 2022). Of concern, however, was that the individuals in the doxycycline arms of these studies consumed between 4 and 16 doses of 200 mg doxycycline per month (Molina et al., 2018; Luetkemeyer et al., 2022). This consumption is 170- to 680-fold higher than the mean population consumption of tetracyclines in European countries in 2021.^1^ It is unknown if the consumption of this quantity of doxycycline in an intermittent fashion could result in antimicrobial resistance (AMR) to tetracyclines and other antimicrobials (Kong et al., 2023; Vanbaelen et al., 2023). A recent systematic review of the effects of oral tetracycline on AMR reported increases in tetracycline-resistant *E. coli* in the gastrointestinal tract, Streptococcus strains in the mouth and respiratory tract pathogens (Truong et al., 2022).

Resistance to tetracyclines can emerge via a number of mechanisms (Grossman, 2016; Kong et al., 2023). Decreased entry via mutations in porin proteins or increased activity of efflux pumps are two important mechanisms (Grossman, 2016). The efflux pumps expel a number of antibiotics, including tetracyclines, out of the cell, making them resistant to these drugs (Grossman, 2016). The expression of these pumps is typically regulated by a number of activating and repressing factors, some of which have been found to be inducible by tetracyclines (Grossman, 2016). An important class of these efflux pumps are the RND-type efflux pumps, such as AcrAB-TolC in *E. coli* and Klebsiella, which confer multi-drug resistance to several different antimicrobial classes, including tetracyclines, penicillins, macrolides, fluoroquinolones, phenicols, and rifampicin (Bialek-Davenet et al., 2011; Grossman, 2016).

The ribosomal protection mechanism is another important resistance mechanism coded by specific tet genes such as tet(M) and tet(O) (Grossman, 2016). These homologs of EF-Tu/EF-G GTPase proteins bind to the h34 site on the ribosome, displacing the tetracyclines bound to it (Grossman, 2016).

Mutations at specific sites of the 30S ribosomal subunit and 16S rRNA are additional resistance mechanisms (Grossman, 2016). Finally, enzyme inactivation of tetracyclines can occur in anaerobes like *Bacteroides fragilis* which comprise part of the human intestinal flora. The gene products responsible for enzymatic inactivation include tet(X), tet(34), tet(37) (Grossman, 2016).

Two of the doxycycline PEP studies evaluated the effect of doxycycline on tetracycline resistance in *Neisseria gonorrhoeae*. Both the studies found no statistically significant effect, but the numbers of gonococcal isolates were extremely low [n = 9 (Molina et al., 2018) and n = 47 (Luetkemeyer et al., 2022)]. Neither study has, as yet, published results of the effect of doxycycline PEP on AMR in any other species.

This provided the motivation for the two objectives of this study. Our first objective was to establish the order in which doxycycline resistance emerged in four target species during passage under doxycycline selection pressure – *Escherichia coli*, *Klebsiella pneumoniae*, *Neisseria gonorrhoeae* and *Neisseria subflava*. We found that resistance first emerged in *K. pneumoniae*, a common colonizer of the gastrointestinal tract that is also a key amplifier and spreader of clinically important AMR genes (Wyres and Holt, 2018). In our second objective, we used a *Galleria mellonella* model of chronic *Klebsiella pneumoniae* infection to interrogate the effect of intermittent exposure to doxycycline on the emergence of doxycycline resistance *in vivo*.

## Materials and methods

### Bacterial strains and growth conditions

Four bacterial species (*Escherichia coli*, *Klebsiella pneumoniae*, *Neisseria gonorrhoeae* and *Neisseria subflava*) with doxycycline minimal inhibitory contentrations (MICs) less than 4 μg/mL were selected from our collection of clinical isolates at the Institute of Tropical Medicine, Antwerp. For *N. gonorrhoeae*, three strains were selected - two from the WHO reference panel (WHO-F and -P) and a circulating strain (Unemo et al., 2016). Detailed information on the six bacterial strains used in this study are provided in Table 1.

**Table 1:** Bacterial strains used in this study.

Organism	Isolate Number	Doxycycline MIC	Clinical origin/Reference
*K. pneumoniae*	M17125	2	Human clinical isolate from ITM collection
*E. coli*	ATCC 25922	2	Human clinical isolate from ATCC collection
*N. subflava*	790/2	1.5	Clinical pharyngeal isolate from an asymptomatic man Laumen et al. (2022)
*N. gonorrhoeae*	WHO-P	1.5	WHO reference strain [11]
*N. gonorrhoeae*	WHO-F	0.25	WHO reference strain [11]
*N. gonorrhoeae*	M22597	3	Clinical urethritis isolate

### *In vitro* induction of doxycycline resistance

The direct colony suspension method was used for inoculum preparation wherein colonies were selected from an 18–24 h (h) BBLTM blood agar (BA) plate. The turbidity of the bacterial suspensions were adjusted to 0.5–1.0 McFarland (McF) standard in phosphate buffer saline (PBS), and replated onto BDTM Chocolate (Choc) agar plates for *K. pneumoniae*/*E. coli* and on BD BBLTM Chocolate II agar (GC II agar with hemoglobin and IsoVitalexTM) for *N. gonorrhoeae*/*N. subflava*.

A doxycycline gradient Etest ranging between 0.016 μg/mL and 256 μg/mL (BioMérieux, France) was placed on all the plates. After overnight incubation at 36°C at 5 (v/v)% CO2, the MIC was noted. A standard loopful of culture (5 mm) was taken from the margin of growth from the most resistant colonies, following the protocol of Wadsworth et al. (Balduck et al., 2022; Raisman et al., 2022). This growth was then suspended in PBS and re-inoculated on a fresh BD BBLTM Chocolate II agar plate (GC Choc) and a new doxycycline *E*-test was placed. The above process was repeated every 24 h for each isolate for a total of 7 consecutive days. Control experiments with each isolate were conducted by passaging the isolate according to the above protocol except that no Etest strip was placed. The experiments were conducted in triplicate.

### *Galleria mellonella* infection model of *Klebsiella pneumoniae*

#### Preparation of live microbial inoculum for infection

The *K. pneumoniae* M17125 isolate was cultured from frozen stocks onto (BA) plate for ≤16 h at 37°C with 5% (v/v) CO2. Single colonies were plated onto fresh Choc agar plates, which were incubated at 37°C with 5% (v/v) CO2 for 6 h. The cultures from the agar plates were suspended in PBS and inoculated into the haemocoel of the *G. mellonella* larvae at a concentration of 104 CFU/larva. This dose of *K. pneumoniae* was determined based on previous experiments that established a dose that enabled the recovery of the bacteria up to 5 days post inoculation with a low mortality rate of the larvae (data not shown).

### *Galleria mellonella*-equivalent dose of doxycycline 200 mg and 100 mg

The doses of doxycycline (Sigma-Aldrich) used were the equivalent of 200 mg (3.333 mg/kg) and 100 mg (1.666 mg/kg) per day used for humans (Wei et al., 2017; Andrea et al., 2019; Khalil et al., 2019). We used larvae with a mean weight of 370 mg (range 300 to 450 mg). This weight was used to calculate the 200 mg-equivalent dose of doxycycline injected into each larva (1.23 ng in 10 μL PBS).

### Injection of *Galleria mellonella* larvae

Last larval stage *G. mellonella* (Terramania, Arnhem, NL) were used for the experiments. The larvae were not fed during the experiment. Only macroscopically healthy, non-discolored larvae were selected. The larvae were placed into individual sterile Petri dishes in groups of 10 per Petri dish. The larvae were kept in an incubator at 37°C with a 5% (v/v) CO2 atmosphere for the length of the experiments. Each control and experimental group consisted of at least 30 larvae.

The larvae were injected in the last pro-legs with 10 μL of various doses of doxycycline/bacteria using 0.3 mL U-100 insulin syringes (BD Micro-Fine). One syringe and needle was used for 10 larvae in each Petri dish.

### Three test groups were evaluated

Group 1 (DoxyPEP): 104 CFU *K. pneumoniae* inoculum followed 10 min later and every 48 h with human PEP equivalent dose of doxycycline – 1.23 ng in 10 μL PBS.

Group 2 (0.5xDoxyPEP): 104 CFU *K. pneumoniae* followed 10 min later and every 48 h with 50% of a human PEP equivalent dose of doxycycline. 0.615 ng in 10 μL PBS.

Group 3 (Control): 104 CFU *K. pneumoniae* inoculum followed 10 min later by 10 μL PBS.

These experimental groups were designed to evaluate two scenarios. Firstly, could the equivalent of 200 mg doses of doxycycline every 48 h induce doxycycline resistance? Secondly, could 50% of this dose induce resistance? – for example, in individuals who acquired *K. pneumoniae* a few hours after taking the 200 mg doxycycline. In pilot experiments we established that the 200 mg equivalent dose of doxycycline was not toxic to the G. mellonella (data not shown).

### Individual- versus network-level induction of AMR

These experimental groups only assess the acquisition of AMR within individuals taking doxycycline PEP. There are a number of population-level mechanisms whereby intense antimicrobial consumption can translate into AMR (Lipsitch and Samore, 2002; Kenyon and Schwartz, 2018). For example, intermittent doxycycline consumption may induce partial resistance in *K. pneumoniae* in one individual. The partial resistance *K. pneumoniae* may then be transmitted (via sex or physical contact) to another individual who is also taking intermittent doxycycline PEP where high level resistance is then induced. To mimic/assess this pathway, 10^4^ CFU of *K. pneumoniae* from randomly selected single colonies obtained from each of the above groups on days 2 and 3 were injected into 5 new larvae ensuring that each larva received a single clone of *K. pneumoniae*. These larvae were then all treated with a human PEP equivalent dose of doxycycline (200 mg) 15 min after the receipt of the *K. pneumoniae*. Isolates from these experiments were termed the day 2 and 3 network-level isolates.

### Retrieval of *Klebsiella pneumoniae* from *Galleria mellonella*

At 24 h after the injection of the bacteria and 24-hourly intervals thereafter, four larvae from each group of 30 larvae were randomly selected for extraction of hemolymph. This was continued for the duration of the experiments – 4 days. The larvae were immobilized by placing them at −80°C for 60 s. They were then placed on a Petri dish, and an incision was made between the two segments closest to the tail of the larva (Dijokaite et al., 2021). Haemolymph was then extracted by squeezing the haemolymph into 1.5 mL centrifuge tubes containing 50 μL PBS, vortexed and divided onto two plates: Klebsiella ChromoSelect Selective Agar (KCA; Merck [Darmstadt, Germany]) with 4 μg/mL doxycycline and KCA without doxycycline. The plates were then incubated at 37°C with a 5% (v/v) CO_2_ atmosphere for 24 h and the number of purple-magenta *K. pneumoniae* colonies were counted. At random, four purple-magenta colonies from the plates with doxycycline per experimental condition were selected for further identification via MALDI-TOF. The method used for MALDI-TOF-MS-based species identity is detailed elsewhere (Laumen et al., 2022). The doxycycline MIC was determined using Etest. If no purple-magenta colonies emerged on the doxycycline plates per condition, then a random selection of 4 purple-magenta colonies from the plates without doxycycline was subjected to MALDI-TOF and the MIC was determined.

Cross-resistance testing for other antimicrobials was carried out using Etest (BioMérieux, France) for all the colonies (*n* = 4) of *K. pneumoniae* that were obtained from the final day of each experimental condition as well as the parental strains for ceftriaxone, ciprofloxacin and azithromycin antimicrobials. The Etests were performed on BDTM Mueller-Hinton agar plates incubated for 16–18 h at 37°C with a 5% (v/v) CO2 atmosphere. All tests were carried out in compliance with the manufacturer’s instructions.

At the end of each experiment both the surviving and dead *G. mellonella*, were kept at −80°C overnight to sedate them. They were then autoclaved at 121°C for 15 min and discarded.

### Whole genome sequencing and bioinformatic analyses

Six strains of *K. pneumoniae* were selected for whole genome sequencing (WGS). These were the parental strain as well as a random selection of 5 strains from the final day of the network selection experiment (Supplementary Table 1). The bacterial isolates were outsourced to Eurofins, where total DNA was extracted followed by library preparation with Stranded TruSeq DNA library preparation kit from Illumina. Sequencing of paired-end reads 2 × 150 bp were performed on NextSeq6000, v2 Illumina platform (Illumina Inc., San Diego, CA, United States) The sequencing data from this study is available under BioProject ID PRJNA949453.

Initial quality control (QC) of the raw reads was carried out using FastQC (Andrews, 2015). To assemble the genome, sequences were first trimmed using trimmomatic (v0.39) and then *de novo* assembled using SPAdes v3.14.0 (Bankevich et al., 2012; Bolger et al., 2014). Once assembly was complete, Quast (v5.0.2) was used to assess the quality of the genome assembly. Assembled scaffolds were annotated using Prokka v1.14.6 (Gurevich et al., 2013; Seemann, 2014).

Accurately identifying genetic organization of genes associated with resistance and single nucleotide polymorphisms (SNPs) is key to understanding the emergence of resistance. Using CLC genomics workbench (v20, CLC bio, Denmark), reference mapping was done, and SNPs were extracted.

### Data analysis

Statistical analyses were conducted using GraphPad Prism® with the Mann–Whitney test used to compare groups. A *p*-value <0.05 was considered statistically significant.

## Results

### *In vitro* induction of doxycycline resistance

**Figure 1:**
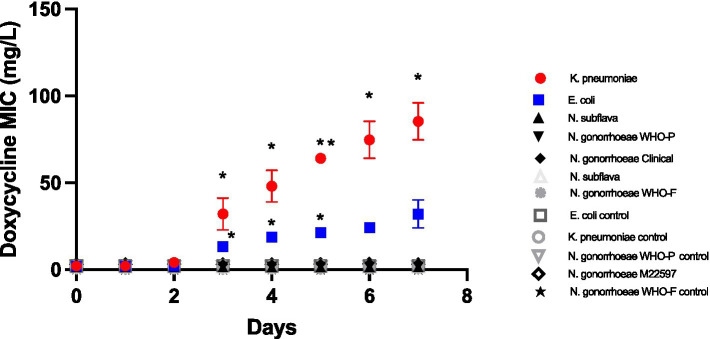
Increase in doxycycline MICs in *Klebsiella pneumoniae*, *E coli*, *N. subflava* and two strains of *Neisseria gonorrhoeae* during passage on chocolate agar plates containing a gradient of doxycycline (0.016 μg/mL to 256 μg/mL). Symbols represent the mean MIC at each timepoint, and the error bars show the standard deviation of the mean. Unpaired *t*-test was done to compare the MICs between controls and doxycycline exposed strain at each timepoint. *p < 0.01; **p < 0.001.

Doxycycline selection led to significant increased doxycycline MICs in *E. coli* and *K. pneumoniae* from day 3 onwards (Figure 1). By day 7 the *K. pneumoniae* MIC had increased from 2 mg/L to a median of 96 mg/L (IQR 64–96; *p* < 0.01), whereas the *E. coli* increased to a somewhat lower median MIC of 24 mg/L (IQR 24–48; p < 0.01 at days 3–5). No increases in MIC were evident in the Neisseria spp. isolates (Figure 1).

### *In vivo* induction of doxycycline resistance in *Galleria mellonella*

#### Individual-level selection

**Figure 2:**
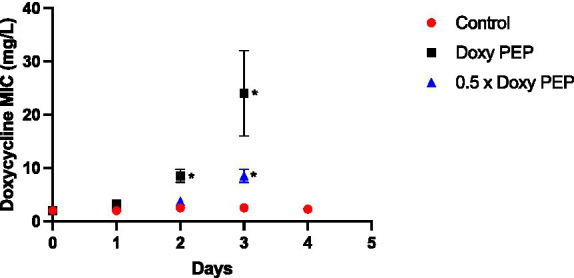
Individual-level selection. Increase in doxycycline MICs in *K. pneumoniae* during individual-level selection following PEP equivalent doses of doxycycline (200 mg/day, Doxy PEP) or 50% of this dose (0.5 x Doxy PEP) in a *Galleria mellonella* model of *K. pneumoniae* infection. Symbols represent the mean MIC at each timepoint, and the error bars show the standard deviation of the mean. Unpaired t-tests were done to compare the MICs between controls and doxycycline exposed strains at each timepoint. *p < 0.01.

In this experiment, a dose of doxycycline was administered to the larvae at baseline and every 48 h thereafter. No *K. pneumoniae* colonies were observed on the doxycycline plates from the larvae at 24 h (Day 1; Figure 2). The first *K. pneumoniae* colonies to emerge on the plates with doxycycline were from the larvae at day 2/48 h (i.e., before they had received their second dose of doxycycline). In the larvae that were exposed to the equivalent of 200 mg doxycycline (DoxyPEP)/0.5 x DoxyPEP, the doxycycline MIC increased from 2 mg/L to a median of 8 mg/L (IQR 7.5–9 mg/L; *p* = 0.013)/4 mg/L (IQR 3.75–4 mg/L; *p* = 0.011), respectively (Figure 2).

Following the receipt of the second dose of doxycycline at day 2, the MICs increased further in the samples obtained the following day, i.e., day 3, to a median of 24 mg/L (IQR 20–28 mg/L; *p* = 0.029) and 8 mg/L (IQR 7.5–9 mg/L; p = 0.013) for the DoxyPEP and 0.5 x DoxyPEP groups, respectively. After this timepoint, *K. pneumoniae* was only cultured from the control group at day 4 when its MIC remained unchanged from baseline (Figure 2). No *K. pneumoniae* from the control group were isolated on the doxycycline plates.

#### Network-level selection

**Figure 3:**
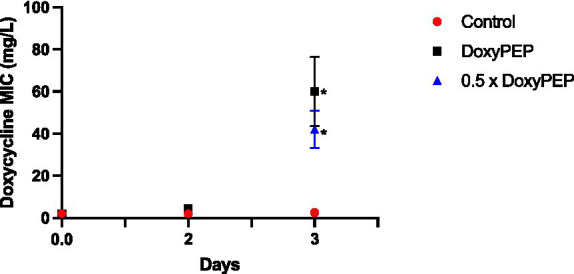
Network-level selection. Increase in doxycycline MICs in *K. pneumoniae* during network-level selection following doxycycline PEP equivalent doses of doxycycline in a *Galleria mellonella* model of *K. pneumoniae* infection. Symbols represent the mean MIC at each timepoint, and the error bars show the standard deviation of the mean. Unpaired *t*- tests were done to compare the MICs between controls and doxycycline exposed strains at each timepoint. *p < 0.05.

To assess network-level selection, *K. pneumoniae* isolates obtained from the larvae in the individual-level experiment on days 2 and 3 were injected into new larvae, followed by a 200 mg equivalent dose of doxycycline. No significant increase in doxycycline MIC was evident on day 2 of the experiments. But the day 3 experiments demonstrated an increase in median MIC to 40 mg/L (IQR 30–52; p = 0.013) and 64 mg/L (IQR 52–72; *p* = 0.014) for the DoxyPEP and 0.5 x DoxyPEP groups, respectively (Figure 3).

#### Cross resistance to ceftriaxone, ciprofloxacin, and azithromycin

The four colonies of *K. pneumoniae* that were obtained from the final day of each experimental condition had elevated ceftriaxone, ciprofloxacin and azithromycin MICs compared to the parental strains. There was no difference in the final MICs between the individual and network conditions. Ceftriaxone MICs increased from a median of 0.064 mg/L (IQR 0.064–0.064 mg/L) to a median of 0.38 mg/L (IQR 0.205–0.470 mg/L; *p* < 0.0001). Likewise, ciprofloxacin MICs increased from a median of 0.064 mg/L (IQR 0.064–0.094 mg/L) to a median of 0.38 mg/L (IQR 0.25–0.470 mg/L; p < 0.0001). The median azithromycin MIC increased from 96 mg/L (IQR 96–96 mg/L) to 192 mg/L (IQR 144–256 mg/L; p < 0.0001).

#### Increased doxycycline MICs associated with mutations in RamA and Rfr-2

**Figure 4:**
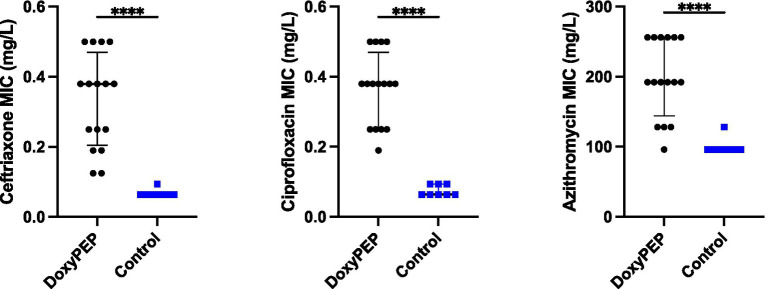
Selection of resistance to ceftriaxone, ciprofloxacin, and azithromycin in *Klebsiella pneumoniae* in *Galleria mellonella* exposed to doxycycline PEP (combined individual– and network-level experiments).

Of the 5 strains with elevated doxycycline MICs that were sequenced, three acquired nonsynonymous mutations in RamR (Table 2). Two strains acquired the Ile26 frameshift mutation, whereas the third strain acquired a Tyr47 frameshift mutation. The two other strains acquired Arg5Cys and Ile74Ser mutations in RrF-2 (Table 2; Figure 4).

**Table 2:** Mutations detected in *Klebsiella pneumoniae* isolates with elevated doxycycline MICs in network experiments.

Strain ID	Doxycycline MIC	Ciprofloxacin MIC	Ceftriaxone MIC	Azithromycin MIC	Gene	Mutations detected	Amino acid change
KPZ_WT	2	0.064	0.064	96	–	–	–
KPZ13_2	64	0.38	0.5	>256	ramR	77_78delTA	Ile26fs
KPZ14_3	64	0.5	0.38	192	rrf-2	C13T	Arg5Cys
KPZ15_2	64	0.38	0.5	>256	ramR	77_78delTA	Ile26fs
KPZ17_2	32	0.5	0.125	96	rrf-2	T221G	Ile74Ser
KPZ18_2	96	0.25	0.38	>256	ramR	132_139delCGCTGTTT	Tyr47fs

fs – frameshift mutation; MIC (mg/L).

## Discussion

In a novel *in vitro* model of doxycycline PEP, we found that doxycycline use resulted in rapid increase in doxycycline MICs in *E. coli* and *K. pneumoniae* but not in *N. subflava* and *N. gonorrhoeae*. Individual-level selection within G. mellonella resulted in increased doxycycline MICs but to a slightly lesser extent than network-level selection. Of particular concern, these increases in doxycycline MICs were associated with increases in MICs for the other three antimicrobials assessed (ceftriaxone, ciprofloxacin and azithromycin).

These findings could be parsimoniously explained by mutations in ramR and rfr-2 (Figure 4). A number of studies have found that a number of mutations (insertions, deletions and point mutations) in ramR are responsible for tetracycline resistance in both clinical isolates and induced-resistant isolates of *K. pneumoniae*. RamR exerts this effect via increasing the expression of the AcrAB efflux pump (Hentschke et al., 2010; Bialek-Davenet et al., 2011, 2013; Villa et al., 2014; Wang et al., 2015). Rrf-2-transcriptional regulator is a transcriptional regulator immediately upstream of a component of the AcrAB pump – oqxB20-RND-efflux-pump (Yen and Papin, 2017). Importantly, we did not attempt to establish experimentally if these mutations we detected are causally related to the changes in MICs we found.

There are a number of other important limitations to this analysis. In the *in vitro* experiments, we only evaluated four bacterial species, while in the *in vivo* experiments, we examined one species. In humans, the rate of intestinal *K. pneumoniae* colonization has been found to be 5 to 25% (Martin et al., 2016; Gorrie et al., 2017) which may mean it would be placed under less selection pressure than a species such as *E. coli* where the carriage is close to universal (Tenaillon et al., 2010; Martinson and Walk, 2020). Our use of doxycycline PEP every 48 h may also only apply to a small proportion of PEP users (Molina et al., 2018, 2023). On the other hand, the experiments were conducted for 4 to 7 days, whereas doxycycline PEP will likely be used in individuals and populations for years to decades. The G. mellonella infection model involves colonization of the hemolymph and not the gastrointestinal tract, which is the typical colonization site for humans. As a consequence, our experimental model likely offers less opportunities for the uptake of resistance-genes from other bacteria via horizontal gene transfer. G. mellonella infection models based on hemolymph infection, including those for *K. pneumoniae* have been shown to provide virulence and therapeutic efficacy results that closely replicate those found in mammals (Wand et al., 2013, 2015; Maguire et al., 2016; Bruchmann et al., 2021). Nonetheless, the large differences between G. mellonella and *Homo sapiens* mean we cannot infer that because doxycycline resistance emerged in the former that it would emerge in the later. We can only conclude that resistance emerged in our model of doxycycline PEP and that this suggests the need for further studies in humans.

We were also unable to evaluate certain indirect pathways whereby doxycycline PEP could select for resistance to tetracyclines and other antimicrobials. Recently, a number of studies have expressed concern that cross-resistance to multiple antimicrobials in various bacterial species may mean that the widespread use of doxycycline PEP will indirectly select for resistance to other antimicrobials (Vanbaelen et al., 2023). Selection of AMR in *N. gonorrhoeae* has frequently been via the selection of clones with resistance to multiple antimicrobials (Sánchez-Busó et al., 2022; Vanbaelen et al., 2023). Gonococcal resistance to tetracyclines is typically caused by the acquisition of the tet (M) gene and/or mutations in rpsJ or porB (Unemo et al., 2016). The intensive use of doxycycline as PEP could provide a selective pressure for the emergence and spread of any or all these mechanisms. This effect would likely be most marked in sexual networks with high rates of partner change and hence a high equilibrium prevalence of *N. gonorrhoeae* (Kenyon and Schwartz, 2018) and intense usage of doxycycline PEP. Intensive consumption of doxycycline in these settings could directly select for these tetracycline resistance associated mechanisms (Vanbaelen et al., 2023). The fact that multidrug resistant clones of *N. gonorrhoeae* are typically resistant to tetracyclines means that doxycycline PEP may inadvertently select for resistance to other antimicrobials (Vanbaelen et al., 2023; Whiley et al., 2023). A similar clustering of resistance to tetracyclines and other antimicrobials has also been shown to pertain to a range of other pathogens such as *K. pneumoniae* and *Staphylococcus aureus* (Gestels et al., 2023). Likewise, a study from France has recently found that 87% of extensively resistant *Shigella sonnei* isolates were doxycycline resistant and likely disproportionately from MSM (Lefèvre et al., 2023). The authors raised the concern that doxycycline PEP may add a further selection advantage to these highly resistant isolates. In a similar vein, we did not evaluate population level selection of AMR. A previous study of minocycline PEP following sexual exposure to *N. gonorrhoeae* found that PEP completely prevented infection with highly susceptible isolates but had no effect on preventing infection with resistant isolates. The authors concluded that at a population level, the widespread use of minocycline PEP would likely select for AMR and was thus not advisable (Harrison et al., 1979). We did not evaluate this pathway.

Notwithstanding these limitations, our *in vivo* model demonstrated that doxycycline can select for resistance to doxycycline and other classes of antimicrobials. As such, the widespread use of doxycycline PEP could contribute to further emergence and spread of multi-drug resistant (MDR) cases in Gram-negative bacteria such as *Klebsiella pneumoniae*. Our findings thus suggest that clinical studies of doxycycline PEP should evaluate the effect on AMR in a wider array of target bacterial species than those considered up to the present. In particular, the effect on Enterobacteriaceae, such as *E. coli* and *K. pneumoniae* should be included. Finally, the effects should include the induction of cross resistance to other antimicrobials.

## Data availability statement

The datasets presented in this study can be found in online repositories. The names of the repository/repositories and accession number(s) can be found below: NCBI, PRJNA949453.

## Author contributions

CK, TV, and SM-B conceptualized the study. CK and ZG conducted the experiments. BX was responsible for the bioinformatic analyses. CK, BX, and SM-B were responsible for the statistical analyses. All authors contributed to the article and approved the submitted version.

## Conflict of interest

The authors declare that the research was conducted in the absence of any commercial or financial relationships that could be construed as a potential conflict of interest.

## Publisher’s note

All claims expressed in this article are solely those of the authors and do not necessarily represent those of their affiliated organizations, or those of the publisher, the editors and the reviewers. Any product that may be evaluated in this article, or claim that may be made by its manufacturer, is not guaranteed or endorsed by the publisher.

## Footnotes

^1^ https://www.ecdc.europa.eu/en/antimicrobial-consumption/surveillance-and-disease-data/database
